# Review of "Mathematical Techniques in Multisensor Data Fusion" by David L. Hall and Sonya A. H. McMullen

**DOI:** 10.1186/1475-925X-4-23

**Published:** 2005-03-29

**Authors:** Ge Wang

**Affiliations:** 1 Department of Radiology, University of Iowa, 200 Hawkins Drive, Iowa City, IA 52242, USA

Data fusion has been a trend in the field of imaging and signal/image analysis. Although multisensor data fusion is still not regarded as a formal professional discipline, tremendous progress has been made since the publication of the first edition of this book in 1992. With this second edition, the authors have been successful in updating us with state-of-the-art methods and techniques in multisensor data fusion. The book involves both algorithms and software tools, and also covers contemporary subjects like smart agents, cognitive aides, and so on.

This book is written according to the Joint Directors of Laboratories (JDL) data fusion group model. There are five levels in the JDL model. The first level deals with association, correlation, estimation, and identification in the data domain. The second and third levels perform knowledge-based processing and utilize expert systems. The fourth level is focused on process monitoring and optimization. The fifth level is devoted to human computer interaction. Chapter 1 serves as an overview. Then, Chapter 2 introduces the JDL model and associated algorithms. Chapters 3–6 cover processing techniques at level one. Chapter 7 gives methods at levels two and three. Chapter 8 targets the control of sensor and information resources at level four. Chapter 9 is for data fusion at level five. Chapter 10 discusses implementation of fusion systems. Chapters 11 and 12 describe emerging applications and information management. The book contains 100 equations, 75 illustrations and key references. The typesetting quality is generally excellent but it would be better if some labels in the figures have been put in larger size. Note that the additons in this book include materials on fusion system control, DARPA's TRIP model, and applications in data warehousing, medical equipment, and defense systems.

Hall (associate dean for research, Pennsylvania State University School of Information Sciences and Technology) and McMullen (captain, US Air Force) are well known experts in the field, and should be congratulated for accomplishing such an excellent job in summarizing the up-to-date essential ideas and results on multisensor fusion. Overall, the book is very informative and not difficult to read for electrical and computer engineers as well as technical managers. These types of practitioners can gain solid advice from the book regarding selection of data fusion methods, balance of trade-offs among commercial off-the-shelf tools, development of multisensor data fusion systems and their applications to solve real-world problems. However, to build a sophisticated data fusion system one may need other references for more comprehensive mathematical theories, more rigorous statistical treatments, and more technical details. The cited literature in the book represents appropriate pointers for that purpose. A good example for further reading is the Handbook [1].

As an engineer in biomedical imaging, I would like to underline the relevance of this book to research on systems biomedicine. Given the completion of the Human Genome Project [2] and the momentum of the Physiome Project [3], in 2003 the NIH defined its Roadmap to guide our efforts towards most important biomedical research, including studies on biological building blocks, pathways, and networks using molecular libraries, imaging, bioinformatics and computational biology. Consequently, multi-modality signal sensing and imaging plays a critical role, which often necessarily generates heterogeous sparse/huge datasets that are multi-dimensional, multi-spectral, dynamic, noisy and/or incomplete. The data fusion techniques discussed in this book are closely related to that for biomedical image registration/fusion. Major challenges and new opportunities are enormous ahead of us. In that context, I hope this book will have a significant impact on the biomedical imaging areas, and recommend it to biomedical imaging researchers in particular and other interested engineers in general.
